# The scaffold of neutrophil extracellular traps promotes CCA progression and modulates angiogenesis via ITGAV/NFκB

**DOI:** 10.1186/s12964-024-01500-5

**Published:** 2024-02-08

**Authors:** Congyi Zhang, Dehai Wu, Bowen Dong, Guanqun Liao, Yang Yu, Shizhuan Huang, Feng Luo, Bin Zhang, Haotian Wu, Tianwei Li, Dixiang Wen, Sheng Tai

**Affiliations:** 1 Department of hepatic surgery, Second Affiliated Hospital of Harbin Medical University, Harbin, China; 2 Key Laboratory of Precision nutrition and health of Ministry of Education, School of Public Health, Harbin Medical University, Harbin, China; 3 Key Laboratory of Organ Regeneration and Transplantation of the Ministry of Education, The First Hospital of Jilin University, Changchun, Jilin, China; 4 Department of Hepatobiliary Surgery, Foshan Hospital Affiliated to Southern Medical University, Foshan, China

**Keywords:** Cholangiocarcinoma, Neutrophil extracellular traps, Integrin αV, Angiogenesis

## Abstract

Neutrophil extracellular traps (NETs) have garnered attention for their dual role in host defense and tumor promotion. With their involvement documented across a spectrum of tumors, their influence on the progression of cholangiocarcinoma (CCA) is of paramount interest. We employed immunohistochemistry and immunofluorescence to detect NET deposition in CCA tissues. Through in vitro and in vivo investigation, including CCA organoid and transposon-based models in PAD4 KO mice, we explored the effects of NETs on cell proliferation and metastasis. Molecular insights were gained through RNA sequencing, enzyme linked immunosorbent assay, and chromatin immunoprecipitation. Elevated intratumoral NET deposition within CCA tissues was associated with poor survival. The influence of NETs on CCA proliferation, migration and invasion was primarily mediated by NET-DNA. RNA sequencing unveiled the activation of the NFκB signaling pathway due to NET-DNA stimulation. NET-DNA pull-down assay coupled with mass spectrometry revealed the interaction between NET-DNA and αV integrin (ITGAV), culmination in the activation of the NFκB pathway. Furthermore, NET-DNA directly upregulated the expression of VEGF-A in cancer cells. The study unequivocally establishes NETs as facilitators of CCA progression, orchestrating proliferation, metastasis, and angiogenesis through ITGAV/NFκB pathway activation. This novel insight positions NETs as prospective therapeutic targets for managing CCA patients. By implementing a variety of methodologies and drawing intricate connections between NETs, DNA interactions, and signaling pathways, this research expands our comprehension of the complex interplay between the immune system and cancer progression, offering promising avenues for intervention.

**Supplementary Information:**

The online version contains supplementary material available at 10.1186/s12964-024-01500-5.

## Introduction

Cholangiocarcinoma (CCA) is a type of primary liver cancer that arises from the epithelial cells of bile ducts, and accounts for 10% of all primary liver malignancies [1]. The main etiopathogenetic factors of CCA are viral hepatitis infection, cirrhosis, intrahepatic lithiasis, toxic exposures and metabolic abnormalities [2]. Currently, surgical resection is the only curative and feasible treatment for CCA. However, recurrence and metastasis limit the efficacy of surgery [3]. For this reason, the molecular mechanisms related to the recurrence and metastasis of CCA need to be further explored so as to find novel therapeutic strategies.

Previous studies have shown that tumor progression, and immune response were modulated by tumor-infiltrated neutrophils intensively [4]. Neutrophils are not only the most abundant circulating leukocytes, but also the initial line of host response during inflammatory states [5]. The anti-microbial activity of neutrophils is mediated mainly through the following three strategies: phagocytosis, degranulation, and neutrophil extracellular traps (NETs) [6]. NET formation is a response by active neutrophils, which can be induced by a series of stimuli, including lipopolysaccharide (LPS), chemokines, immune complexes and complement [7]. NETs are structures consisting of decondensed DNA scaffold containing proteases, cytokines, and chemokines [8]. Initially, NETs were observed to capture circulating cancer cells with their sticky web-like structure, which favored the process of cancer metastasis [9]. It has been demonstrated that NET-related proteases promoted caner metastasis by destroying endothelial cells contacts [10], or remodeling the extracellular matrix [11]. However, whether NETs exist in CCA and, if so, how NETs will interact with CCA are still unclear.

In the present study, we aimed to investigated the potential impact of NETs in CCA.

## Material and methods

### Patients and tissue samples

Matched CCA and adjacent non-tumor tissues were collected from patients who underwent surgery in the Second Affiliated Hospital of Harbin Medical University (between July 2019 and October 2019). All patients involved in this study provided informed consent. The study was approved by the Research Ethics Committee of the Second Affiliated Hospital of Harbin Medical University (KY2019–046). Detailed clinicopathological features of 7 CCA specimens involved in this study are shown in Supplementary, Table 1.

### Immunohistochemical (IHC) staining and immunofluorescence assay.

The tumor and adjacent non-tumor sections were treated with dewaxing, rehydration, antigen repair and blocking. After that, the sections were incubated with primary antibodies at 4 °C overnight. The following day, for IHC, the sections were incubated with secondary antibodies for 1 hour. Then, sections were stained with diaminobenzidine (DAB kit; Vector Laboratories, Burlingame, CA, USA) and counterstained with hematoxylin (Sigma, St. Louis, MO, USA), according to the manufacturer’s protocol. The staining intensity was scored as 0 (negative), 1 (weak), 2 (moderate) and 3 (strong). The percentage scores were defined as 0, < 5%; 1, 5–25%; 2, 26–50%; 3, 51–75%; 4, > 75%. The histological score for each section was calculated using the following formula: Histological score = proportion score × intensity score. For IF, the sections were incubated with fluorescent secondary antibodies for 1 hour. Then, nuclei were stained with DAPI. Image J software was applied to quantify the integrated fluorescence intensity of the labeled antigens in the acquired images. The mean gray value was calculated using the following formula: Mean gray value = Integrated Density/Area. Information of all the primary antibodies used in this study is provided in Supplementary, Table 2.

### Cell lines and isolation of neutrophils

Human CCA cell lines, RBE and KMBC were obtained from the Chinese Academy of Science (Shanghai, China). The normal primary human biliary epithelial cells (HIBEpiC) were gained from ScienCell Research Laboratories (Carlsbad, CA, USA). Neutrophils were isolated from healthy volunteers’ peripheral blood with Ficoll density gradient (P4937, Sigma-Aldrich), centrifuged at 500 g for 30 mins. All cell lines were cultured in Roswell Park Memorial Institute 1640 (Gibco, USA) supplemented with 10% fetal bovine serum (Gibco, USA) and 1% antibiotics (100 U/ml penicillin and 100 μg/ml streptomycin) at 37 °C, 5% CO_2_.

### NETs induction and purification

Isolated neutrophils were cultured with 500 nM PMA (P8139, Sigma-Aldrich) for 3 hours. After that, the supernatant was removed and the neutrophils were gently washed with 2 ml of frozen PBS. Following this, repeated pipetting up and down with 1.5 ml of frozen PBS was performed to wash away adherent NETs. Then, centrifugation at 1000 g for 15 mins was carried out. The supernatant was collected and the concentration of DNA in NETs was measured using spectrophotometry.

### NET-DNA purification, biotinylation and cell surface protein isolation

A sonicator was used to fragment the NET-DNA to lengths of 500 bp, and a MicroElute DNA clean up kit (D6296, OMEGA) was applied to purify NET-DNA. A DNA labelling kit was employed to biotinylate NET-DNA (89,818, Thermo Fisher Scientific). Pierce cell surface protein isolation kit (A44390, Thermo Fisher Scientific) was applied to isolate cell membrane proteins according to the manufacture’s protocol.

### NET-DNA pull down assay

Biotinylated NET-DNA was incubated with cell membrane proteins at 4 °C overnight, supplemented with protease inhibitor. On the following day, the complex was incubated with streptavidin-agarose beads for 2 hours. The beads were washed with IP washing buffer thrice. The protein-DNA complex was eluted with IP elution buffer. Then, the proteins were separated with silver staining and were analyzed by mass spectrometry.

### MPO-DNA ELISA

To quantify NETs in mouse sera, a capture ELISA myeloperoxidase associated with DNA was prefomed as previously reported [12]. In order to evaluate the circulating nucleosomes derived from NETs, we tested myeloperoxidase attached to nucleosomes. We used the mouse MPO ELISA kit (Hycult biotech, HK210–01) according to the manufacturer’s directions. 100 μl of serum was added to the wells and incubated for 3 hours. After that, each well was washed for 3 times. Then 100 μl incubation buffer containing a peroxidase-labeled anti-DNA mAb (component No.2, Cell Death ELISA^PLUS^, Roche; Cat. No: 11774424001) was addded to each well and incubated for 2 days. The values of NETs formation were determined as the fold change in absorbance.

### Establishment of cholangiocarcinoma organoids

CCA tissues were isolated from patients who were diagnosed with CCA and had undergone surgical treatment at the Second Affiliated Hospital of Harbin Medical University. The tumor tissues were digested to obtain epithelial carcinomatous cells. Isolated tumor cells were mixed with 150 μl Matrigel and incubated at 37 °C until the matrix was solidified; after that complete medium was added. Complete medium consisted of DMEM/F12 supplemented with B27, N2, 1.25 mM N-acetylcysteine, 10 mM HEPES, 10 mM nicotinamide, 10 nM gastrin-I, 10 μM Forskolin, 5 μM A83–01 (TGFβ inhibitor), 3 nM dexamethasone, 100 ng/ml FGF10, 50 ng/ml EGF, 25 ng/ml HGF. Passaging was performed at 1:3 spilt ratios. Precooled DMEM/F12 was used to remove organoids, dissociated into small fragments, and embedded into fresh Matrigel. As for cryostorage, organoids were removed from the matrix, mixed with freezing solution (90% fetal bovine serum and 10% DMSO), and frozen using standard procedures. All the procedures were performed according to approved guidelines.

### Cell counting kit-8 assay

An aliquot of 10^3^ tumor cells was cultured in 96-well plates overnight till the cells were attached. One group was treated with NETs; the other group was treated with PBS. The following day, a solution containing CCK-8 reagent was used to replace the previous medium and cells were cultured for 2 hours. Finally, the absorbance was measured at 450 nm.

### Transwell migration and invasion assay

Migration and invasion were detected by Transwells coated with non-Matrigel or Matrigel. Media containing serum was added to the plate wells. After CCA cells adherence, NETs (5 ng/μl); DNase-I (0.25 mg/ml, Roche, 143,582); BAY11–7082 (5 μM) was added to the upper chamber. After 12 hours, the cells that penetrated into the bottom layer were fixed and stained and counted.

### Chromatin immunoprecipitation (ChIP)

NFκB ChIP assays were carried out according to the manufacturer’s protocols (Millipore, 17–10,085). Briefly, NET-DNA stimulated RBE and KMBC cells for 18 hours. Tumor cells underwent formaldehyde fixation (final concentration was 1%), cell lysis and sonication. After centrifugation, the supernatant was diluted with dilution buffer containing protease inhibitors. The immunoprecipitation were performed using NFκB (1:100) antibody overnight. Samples were washed and incubated with NaCl (5 M) for 4 hours at 65 °C. DNA was recovered with phenol/chloroform extraction and ethanol precipitation. The PCR products were separated by agarose gel electrophoresis. The primers information used for Chip-PCR was listed in the Supplementary Table 3.

### Quantitative real-time polymerase chain reaction (qPCR)

Total RNA was isolated from RBE or KMBC cells with RNA Miniprep Kit (Axygen), and cDNA was synthesized using High Capacity RT Kit. qPCR was performed using SYBR Green on a 7500 Fast PCR System. Then the expression of levels of mRNA normalized to GAPDH. The primers information was listed in the Supplementary Table 3.

### Z-score evaluation of the NETs and angiogenesis

Z-score was applied to mirror the activity of given pathways by integrating feature gene expressions. Gene sets containing the genes referring to NETs initial biomarkers or angiogenesis related markers were subjected to the z-score algorithm implemented in the R package GSVA. The value of each gene set was enumerated as NETs score and angiogenesis score. Related gene sets calculated for Z-score were listed in the Supplementary Table 4.

### Conditioned medium

To prepare conditioned medium (CM), CCA cell lines (2*10^5^cells/mL) were cultured in RPMI 1640 with no FBS for 36 h, and the supernatant was obtained by centrifugation at 12,000 g for 10 min. After centrifugation, the supernatant was used as the CCA cells CM. The CM was stocked at − 80 °C.

### Tube formation assay

HUVECs were purchased from Meisen. Matrigel (BD356234; Corning, USA) was placed on ice, oscillated. 100 μL of Matrigel was added to each well of a precooled 24-well plate. Matrigel was incubated at 37 °C for 45 min until it solidifed. HUVECs were digested with trypsin, centrifuged, resuspended, and counted. The concentration of the cell suspension was adjusted to 3*10^5^cells/mL. A 100 μL cell suspension and indicative conditioned medium was added to each of the wells. The CM and DMEM containing 10% FBS were mixed in a ratio of 2:1 and applied to HUVECs. After incubation at 37 °C, tubules formed after 4 h were photographed using a microscope.

### Animal model

Hydrodynamic liver injection was performed according to previously reported method [13]. Briefly, DNA suspended in 1 ml saline was injected via tail vein for 7–10 seconds into 4-week-old male wild type C57BL/6 mice or PAD4 KO mice. The amount of DNA for each mouse was 22.5 μg for AKT-PT3EF1α, 22.5 μg for YAP-PT3EF1α, 5 μg for pCMV-Sleeping Beauty (SB) transposase. Six weeks later all surviving mice were sacrificed. Male wild-type (C57BL/6) mice were purchased from Charles River (Beijing, China). Peptidyl arginine deiminase type IV (PAD4^−/−^) knockout mice were purchased from GemPharmatech Co. Ltd. (Nanjing, China). Approval from Animal Ethics Committee of Harbin Medical University was taken for conducing the animal experiment (KY2019–046).

## Results

### NETs were detected in CCA tumor tissues and predicated poor prognosis.

**Fig. 1:**
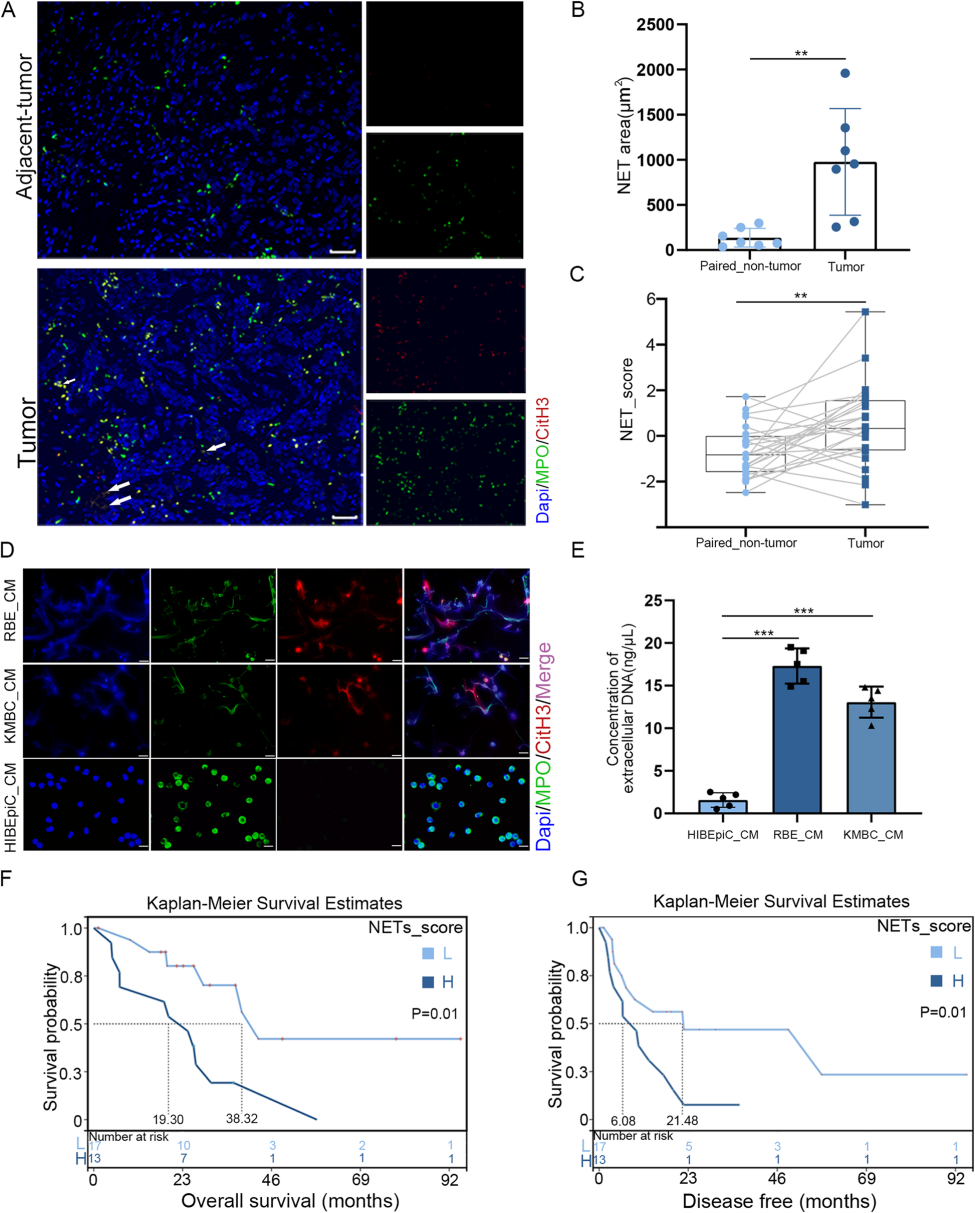
NETs were detected in CCA tumor tissues and predicated poor prognosis. **A** Representative immunofluorescence images of human cholangiocarcinoma tissue sections (n=7) showing increased neutrophil extracellular traps (NETs) formation in tumor compared to non-tumor tissues of the same patient, Dapi (blue), anti-MPO (green), anti-CitH3 (red), Scale bar=50μm. **B** The imaging analysis shows a higher level of NETs in CCA tissues compared to non-tumor tissues. **C** NETs score was analyzed in 27 human CCA from GEO databases (GSE107943). **D** Representative immunofluorescence images of NETs induced by conditioned media derived from each cell lines, Dapi (blue), anti-MPO (green), anti-CitH3 (red), Scale bar=40μm. **E** Quantification of NETs by extracellular DNA concentration. **F** and** G** Kaplan-Meier disease-free and overall survival curves were generated based on the NETs score for human CCA from the GEO database (GSE107943) post-operatively for 92 months, stratifying patients into high (n=15) and low (n=15) NETs score groups. **P<0.01, ***P<0.001. Data are means ± SD of three independent experiments

IF staining of 7 pairs of cholangiocarcinoma tissues and adjacent non-tumor tissues was performed. The IF staining results showed that CitH3, the NETs marker, was positive mostly in tumor tissues (Fig. 1A). In the comparison between these groups, tumor tissues exhibited higher levels of NETs compared to their paired non-tumor tissues (Fig. 1B). We also evaluated the NETs score of paired CCA tissues from GEO databases (GEO107943). The NETs score was significantly increased in tumor tissues compared with that of the paired adjacent non-tumor tissues (Fig. 1C). According to the previous reports, abundant chemotactic factors released by tumor cells were associated with the formation of NETs [14, 15]. To examine whether CCA cell lines induced NETs in vitro*,* neutrophils were cultured with CM from cancer cells including RBE and KMBC. The results indicated that CM from both RBE and KMBC induced NETs formation in vitro (Fig. 1D)*.* The quantification of NETs induced by tumor cells was tested by a NanoDrop spectrophotometer by measuring the amount of extracellular DNA. The results showed that neutrophils released more extracellular DNA after stimulation by CCA cell lines CM (Fig. 1E). These results suggested that NETs were mainly formed in tumor tissues. We then compared the disease-free and overall survival of patients diagnosed with CCA with NETs score above or below median. Patients with above median (high) NETs score had noticeably shorter disease-free and overall survival than patients with below median (low) NETs score (Fig. 1F and G), which was supported by data from GEO database (GSE107943).

### NETs facilitated proliferation and metastasis of CCA cell lines

**Fig. 2:**
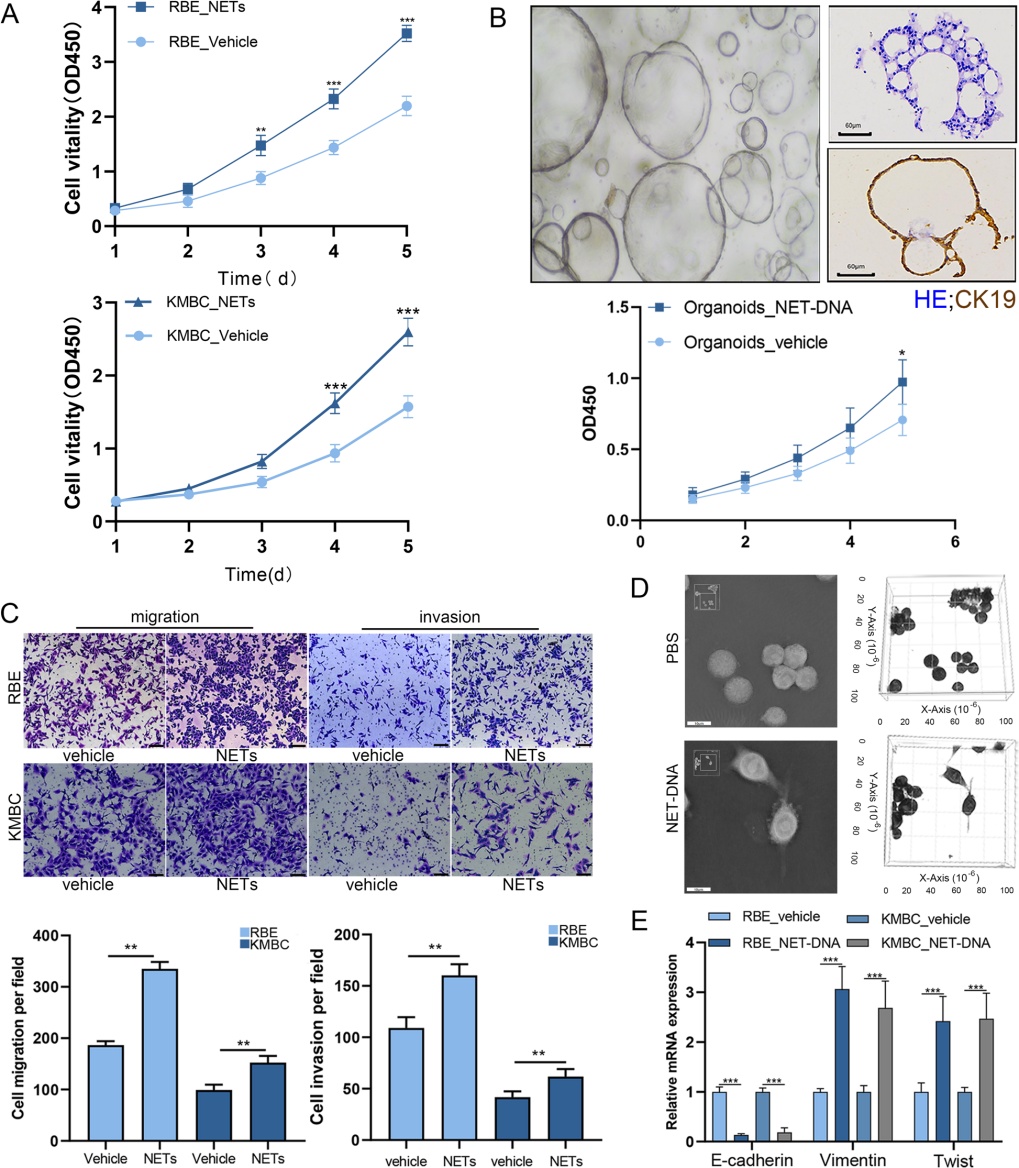
NETs facilitated proliferation metastasis of CCA cell lines. **A** Proliferation rate was analyzed by a CCK-8 assay of indicated CCA cells treated with NETs or vehicle (5ng/μl). **B** Images of isolated cell cluster and growing organoids. Proliferation rate was analyzed by a CCK-8 assay of cholangiocarcinoma organoids cocultured with NET-DNA or vehicle (5ng/μl). **C** Transwell migration and invasion assays for indicated cell lines treated with NETs or vehicle (5ng/μl, 18h). **D** Optical tomography images of morphological changes in RBE cells induced by NET-DNA (5ng/μl, 24h). **E** qPCR analyzed the expression of E-cadherin, vimentin and Twist in RBE and KMBC cells treated with NET-DNA or vehicle (5ng/μl, 24h). Data are means ± SD of three independent experiments. *P<0.05, **P<0.01, ***P<0.001

To detect the role of NETs in CCA, we isolated neutrophils from healthy volunteers and induced NETs formation in vitro (Fig. S1A and B). We found that NETs efficiently strengthened the proliferation of RBE and KMBC cells compared to the vehicle group (Fig. 2A). CCA organoids were established in vitro, and the CCK-8 assay demonstrated that incubation with NETs could increase the proliferation of CCA organoids (Fig. 2B). Transwell migration and Matrigel invasion assay showed that NETs treatment increased the migratory and invasive capabilities of RBE and KMBC cells (Fig. 2C). NETs contain many types of proteins, proteases, cytokines and DNA scaffoldings. In order to elucidate which component of NETs played a vital role in promoting CCA progression, we treated NETs with DNase-I, which degraded NET-DNA to obtain NET-proteins. In addition, we also extracted NET-DNA from neutrophils co-cultured with PMA. Then, we treated tumor cells with NET-proteins and NET-DNA, separately. The results showed that the proliferation, migration, and invasion of cancer cells were promoted by NET-DNA substantially (Fig. S2A and B). Moreover, the most effective concentration of NET-DNA was found to be 5 ng/μl (Fig. S2C).

Optical tomography images showed that treatment with NET-DNA changed the morphology of RBE cells to spindle shapes (Fig. 2D). Epithelial to mesenchymal transformation (EMT) is a crucial process that enables malignant cells to exhibit migration and invasion abilities. IF results indicated that when RBE cells and KMBC cells were co-cultured with NET-DNA, the expression of E-cadherin (an epithelial marker) was decreased while the expression of vimentin (a mesenchymal marker) was increased (Fig. S2D). Similar results were obtained by PCR (Fig. 2E). Previous study has reported that Snail, Slug and Twist were the main upstream transcription regulators of EMT [16]. Our results revealed that only Twist mRNA levels were upregulated after NET-DNA treatment (Fig. 2E and Fig. S2E). Taken together, these observations suggested that NETs promoted the progression of CCA cell lines through NET-DNA and enhanced the migration and invasion of CCA cells by inducing EMT.

### Tumor progression was attenuated in a NETs-depleted CCA mouse model.

**Fig. 3:**
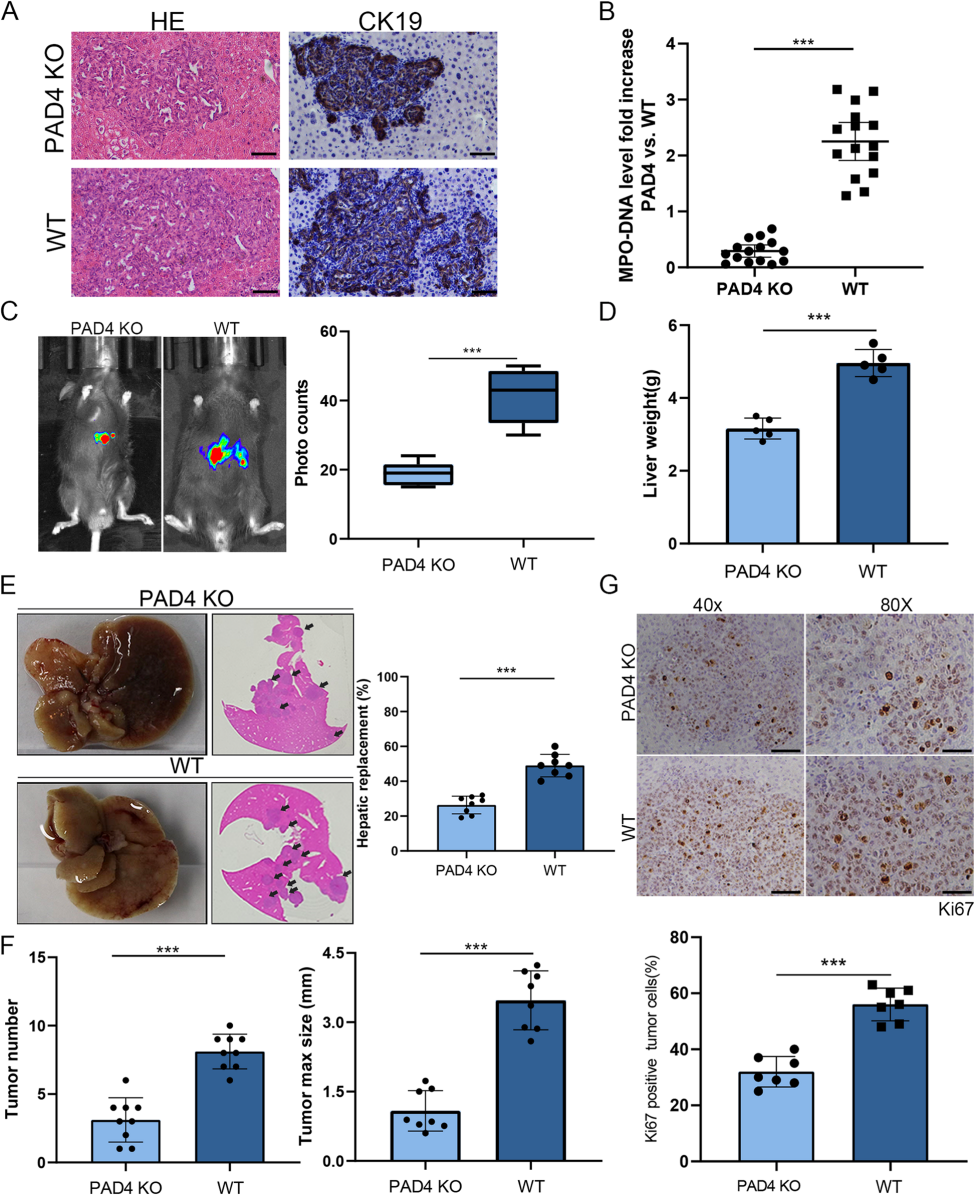
DNase-I alleviated the carcinogenesis of NETs in a CCA mouse model. **A** H&E and CK19 staining of AKT/YapS127 mouse for indicated group, Scale bar=50μm. **B** MPO-DNA levels analyzed by ELISA kit were much higher in WT mice compared with PAD4 KO mice. **C** Representative bioluminescence imaging of the transposon-based CCA model in WT mice and PAD4 KO mice. Photos were taken 21days after the hydrodynamic liver injection. **D** The weight of livers in the indicated group. **E** Representative gross images and HE staining of liver sections from WT mice and PAD4 KO mice. **F **and** G** Quantification of tumor burden in the indicated group, as determined by tumor number, tumor maximum size and Ki67 positive tumor cells. Data are means ± SD of three independent experiments. ***P<0.001

To further confirm that NETs promoted CCA progression in vivo. A CCA mouse model was established by hydrodynamically injecting AKT and YapS127A over-expression plasmids into PAD4 KO mice which were incompetent for NETs formation due to a key enzyme deficiency, and WT C57BL6 mice (Fig. 3A). Agarose gel electrophoresis was applied to confirm the genotype of the mice (Fig. S3A). As expected, the burden of NETs was decreased in PAD4 KO mice compared with that in WT mice, as evidence by MPO-DNA ELISA assay and IF staining (Fig. 3B and Fig. S3B). In addition, bioluminescence imaging and liver weight showed that the CCA progression was slowed down significantly in PAD4 KO mice compared to the wild type mice (Fig. 3C-D). Similar results were observed the tumor grew in PAD4 KO mice were obviously more fewer and smaller than that in WT mice (Fig. 3E and F). Moreover, we found that tumor sections from WT mice presented stronger Ki67 staining than those from PAD4 KO mice (Fig. 3G). These results revealed that NETs depletion effectively inhibited CCA progression.

### NET-DNA promoted the progression of CCA via NFκB signaling pathway

**Fig. 4:**
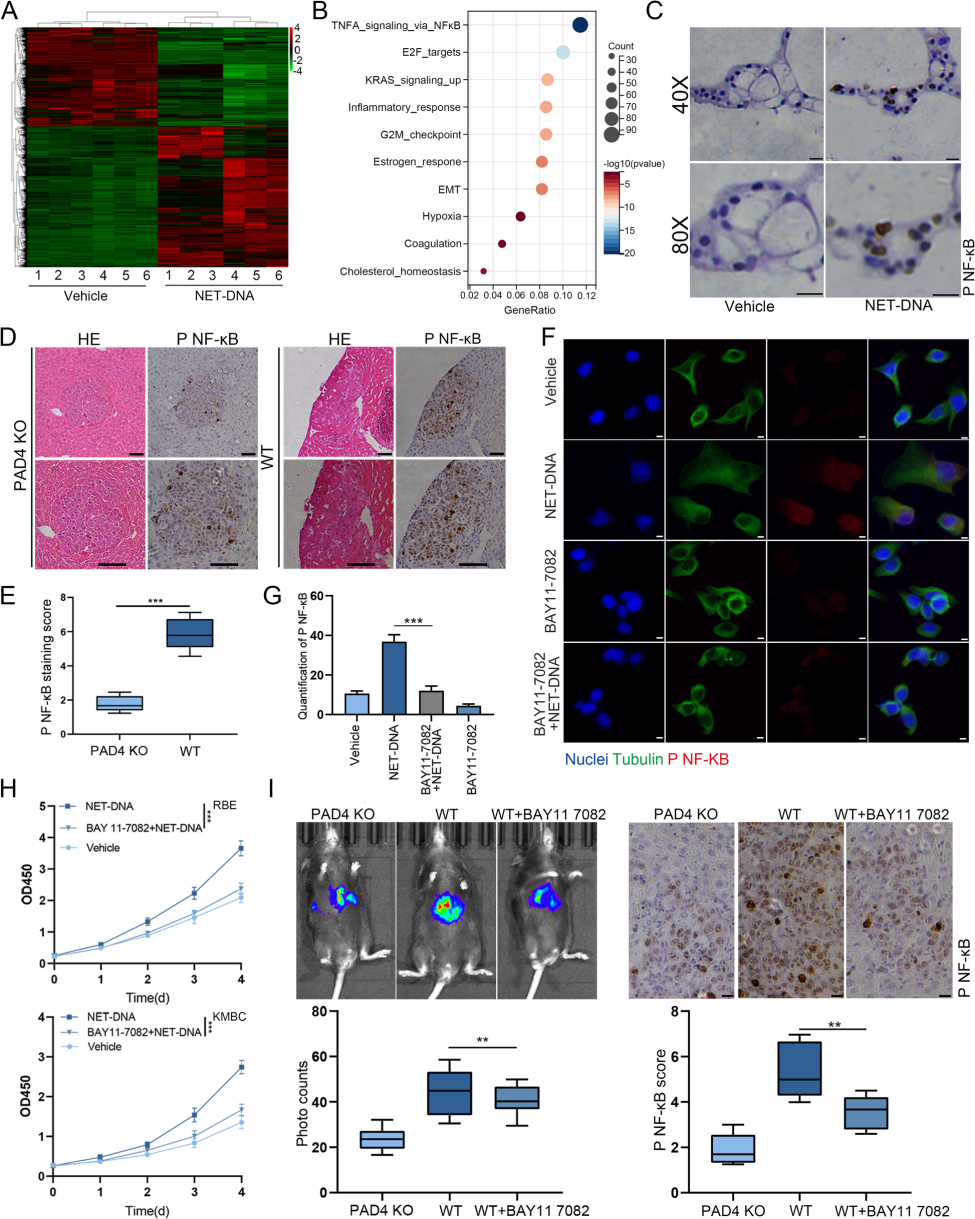
NET-DNA promoted the progression of CCA via NKκB signaling pathway. **A** Heat map of DEGs in CCA cells treated with NET-DNA or vehicle (5ng/μl) for 3 days. **B **KEGG enrichment analysis of most differentially expressed genes (absolute -*log*_*10*_ fold change >2). **C** The expression of p-NFκB was measured by IHC of CCA organoids treated with or without NET-DNA (5ng/μl, 18h), Scale bar=10μm. **D** IHC detection of p-NFκB from liver sections of CCA mouse model in the indicated group, Scale bar=30μm. **E** Quantification of the p-NFκB staining scores in tumor sections (*n*=5) from PAD4 KO and WT mice. **F** IF analysis of p-NFκB (red), tubulin (green) in indicated cells with or without BAY11-7082 treatment (5μM). Scale bar=5μm. **G** Quantification of p-NFκB in the indicative group. **H** Proliferation rate after BAY11-7082 treatment was analyzed by CCK-8 assay of indicated CCA cells. **I** Representative bioluminescence imaging demonstrated that downregulated NFκB signaling pathway with BAY11-7082 (10mg/kg/i.p.) by every 2 days prevented CCA progression induced by NETs, photos were taken 21days after the hydrodynamic liver injection; the statistical analysis of bioluminescence signal in the below panel; IHC analyzed the expression of p-NFκB in the indicative group. Data are means ± SD of three independent experiments. *P<0.05, **P<0.01, ***P<0.001

To investigate the mechanism by which NETs administration promoted proliferation, migration and invasion of RBE and KMBC cells, RNA sequencing was carried out using total RNA extracted from RBE cells treated with NET-DNA or vehicle for 3 days. Impressive alternations in the gene expression profiles of RBE cells were detected (Fig. 4A). Significantly different expressed genes were filtered and enriched in the KEGG signaling pathway. The top 10 signaling pathways were listed and we found that the NFκB signaling pathway ranked as first (Fig. S4A and B). To further confirm if NET-DNA could indeed activate the NFκB signaling pathway, IHC of CCA organoids was performed. The results showed that the expression of p-NFκB was elevated after treatment with NET-DNA (Fig. 4C). In the CCA mouse model, we observed elevated levels of p-NFκB in WT mice, whereas it was relatively low in PAD4 KO mice (Fig. 4D and E). In addition, IHC staining also revealed a positive correlation between the protein expression of p-NFκB and tumor size (Fig. S4B).

BAY11–7082, the novel specific inhibitor of NFκB, was applied to further investigate the mechanisms of NET-DNA induced CCA progression. The expression of p-NFκB induced by NET-DNA stimulataion in RBE cells was attenuated by BAY11–7082 (Fig. 4F and G). The CCK8 assay indicated that BAY11–7082 could inhibit the variation in proliferation of CCA cells induced by NET-DNA (Fig. 4H). Analogously, after treating with BAY11–7082, the invasion and migration abilities of NET-DNA stimulated RBE and KMBC cells were apparently blocked (Fig. S4C). In vivo experiments demonstrated that downregulation of NFκB signaling pathway prevented the progression of CCA induced by NETs (Fig. 4I). The above data suggested that the NFκB signaling pathway is critical for NET-DNA mediated CCA progression.

### NET-DNA mediated NFκB signaling pathway activation via ITGAV

**Fig. 5:**
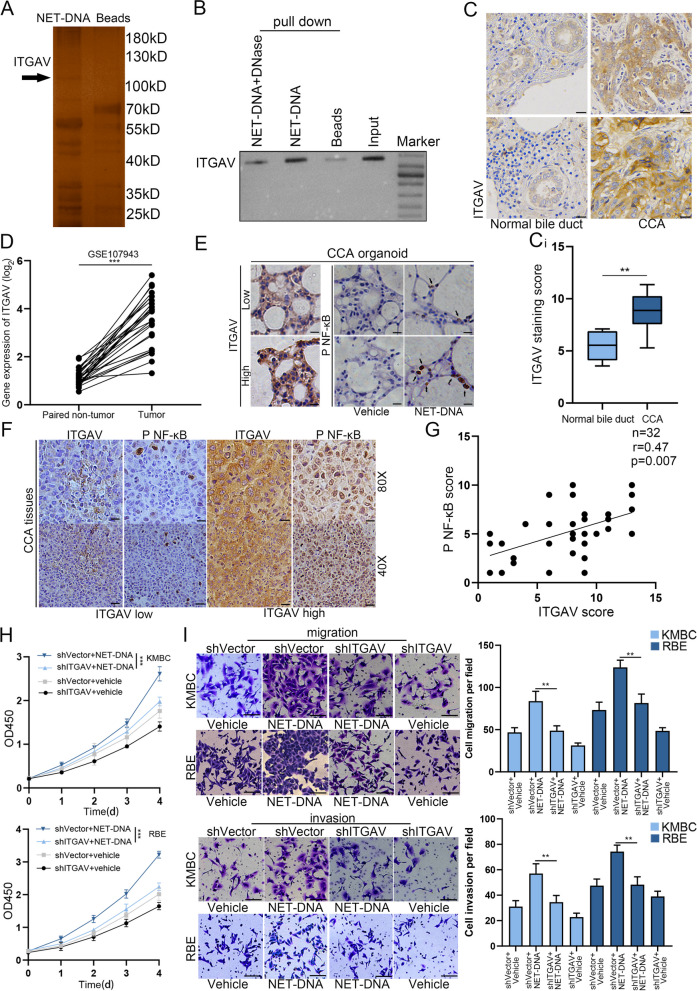
NET-DNA mediated NFκB signaling pathway activation via ITGAV. **A** Membrane protein extracted from RBE cells were incubated either with uncoupled beads or with beads coupled to biotin-NET-DNA. ITGAV, indicated by the arrow. **B** Immunoprecipitated bound proteins were blotted with anti-ITGAV antibody. **C** Representative images of ITGAV detected by IHC in CCA patients. Scale bar=50μm. Ci IHC staining score was used to compare the protein expression of ITGAV between CCA tissues (*n*=100) and the adjacent normal bile duct (*n*=100). **D** GEO databases (GSE107943) showed that mRNA levels of ITGAV were magnificently increased in CCA tissues (*n*=27) compared with the paired adjacent normal tissues (*n*=27). **E** ITGAV overexpression was positively correlated with p-NFκB upregulation in CCA organoid sections, Scale bar=50μm. **F** Representative images of IHC staining analysis of ITGAV and p-NFκB expression in CCA tissues from mouse model. **G** IHC scores were used to exam correlation of protein expression between ITGAV and p-NFκB (*n*=32, *p=*0.007, Spearman’s coefficient r=0.47). **H** Proliferation rate after transfection with short hairpin RNAs was analyzed by CCK-8 assay of indicated CCA cells. **I** Transwell migration and invasion assays for indicated cell lines. Data are means ± SD of three independent experiments. *P<0.05, **P<0.01, ***P<0.001

To identify how NET-DNA interacts with CCA cells to induce NFκB signaling pathway activation, we incubated biotinylated NET-DNA with plasma membrane proteins isolated from RBE cells. The proteins that interacted with NET-DNA were identified by silver staining and mass spectrometry and repeated for three times (Fig. 5A and Fig. S5A). To recognize the potential DNA receptors, we compared the amino acid sequence of pulled down proteins by streptavidin beads attached to biotinylated NET-DNA with that of classical DNA sensor. We detected that the amino acids of ITGAV matched with the DNA binding domain of HMGB1 (Fig. S5B). And the interaction between ITGAV and NET-DNA was confirmed by Western blot (Fig. 5B). We examined a tissue microarray cohort of 100 patients with CCA using IHC staining, the reulsts indicated that ITGAV was highly expressed in human CCA tissues compared to normal tissues, as evidenced by immunohistochemical staining, data from GEO and TCGA database (Fig. 5C-D and Fig. S5C). Next, we analyzed the association between ITGAV and p-NFκB expression in CCA organoids. After co-culture with NET-DNA, the CCA organoids with high levels of ITGAV were predicted to have high levels of p-NFκB (Fig. 5E). Furthermore, consistent results were obtained from IHC staining of liver sections from CCA mouse model (Fig. 5F). IHC staining indicated a positive correlation between the protein expression of ITGAV and p-NFκB in CCA tissues (Fig. 5G).

To comprehend the role of ITGAV in CCA, RBE and KMBC cell lines were transfected with short hairpin RNAs (shRNAs) by lentivirus to silence ITGAV expression. The results indicated that shITGAV-3 exhibited the greatest silencing effect and, therefore, shITGAV-3 was used for immunofluorescence staining (Fig. S5D and E). IF exhibited that ITGAV knockdown in RBE and KMBC cell efficiently abolished the activation of NFκB signaling pathway induced by NET-DNA (Fig. S5F). We also detected that ITGAV knockdown inhibited the proliferation of tumor cells stimulated by NET-DNA (Fig. 5H). Moreover, ITGAV knockdown abrogated the migration and invasion abilities of tumor cells that were induced by NET-DNA (Fig. 5I). Taken together, these data illustrated that NET-DNA promoted the proliferation and metastasis of tumor cells via interaction with ITGAV.

### NET-DNA regulated the production of VEFG-A via ITGAV/ NFκB axis to promote angiogenesis

**Fig. 6:**
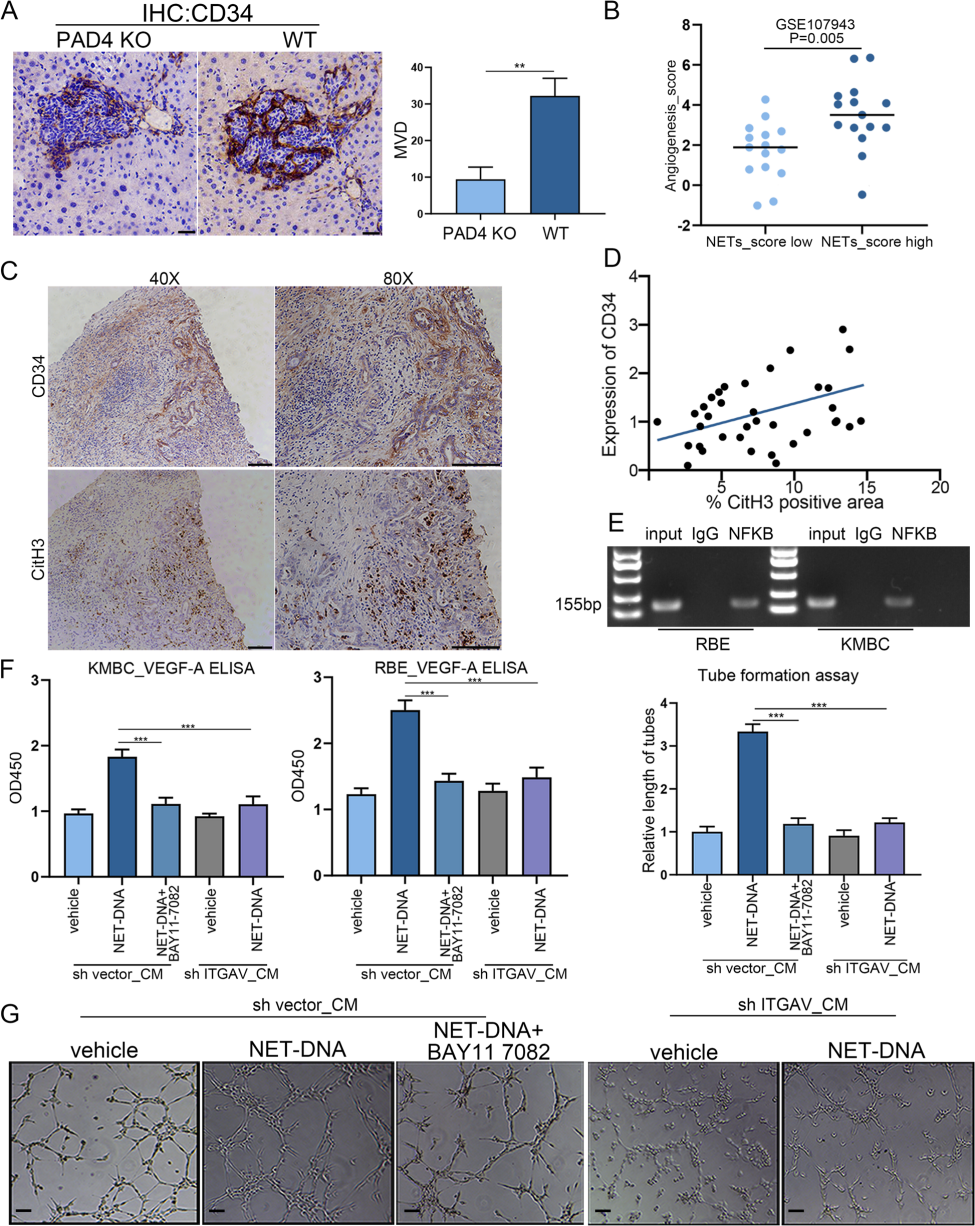
NET-DNA promoted CCA cell lines synthesized VEGF-a via ITGAV/ NFκB axis and induced angiogenesis. **A** Representative images of CD34 staining showed that increased angiogenesis in WT mice group compared to the PAD4 KO mice group. Scale bar=50μm. **B** Elevated angiogenesis_score in high NETs_score group compared to the NETs_low score group. **C** Positive correlation between Cit-H3 and CD34 expression in liver sections from patients with CCA. **D** Correlation of protein expression of CD34 and CitH3 (*n*=37, *p=*0.0077, Spearman’s coefficient r=0.401). **E** Chip-qPCR assay showed NFκB can bind to the VEGF-A promoter regulatory region. **F** VEGF-A ELISA assay showed that NET-DNA stimulated ITGAV/NFκB pathway to induce VEGF-A biogenesis. **G** The conditioned medium from CCA cell lines was employed in a tube formation assay, revealing that NET-DNA promotes angiogenesis through the ITGAV/NFκB pathway. Scale bar=20μm. Data are means ± SD of three independent experiments. **P<0.01, ***P<0.001

Angiogenesis is the growth of new blood vessels, which occurs pathologically during inflammation or tumor growth and physiologically during embryonic development [17]. We analyzed the DEGs involved in angiogenesis and genes tended to be angiogenic functions, including VEGF-A, ANG, ANGPTL4 and YIPF4, were dramatically promoted after NET-DNA stimulation (Fig. S6A). Gene set enrichment analysis (GSEA) revealed that VEGF signatures were enriched in the NETs treatment group (Fig. S6B). We then performed IHC staining, the results showed that a marked decreased expression of CD34 was observed in tumor sections from PAD4 KO mice (Fig. 6A). In addition, no correlation was observed between CD34 staining and tumor size, indicating that a reduction in CD34 in PAD4KO mice was not a reflection of their smaller size (Fig. S6C). We also compared the angiogensis score in CCA patients with low or high NETs score from GEO database (Fig. 6B). Next, we visualized co-localization of NETs and CD34 in tumor sections from CCA patients. CD34 expressed highly in NETs rich areas (Fig. 6C). The IHC results showed that the protein expression levels of CD34 exhibited a positive correlation with CitH3 in CCA tissues (Fig. 6D). To verify the relationship between VEGF-A and NFκB, NFκB specific antibody was applied to performed chip-qPCR indicated that NFκB was recruited to the VEGF-A promoter regulatory region when compared to the IgG group (Fig. 6E). In our investigation of NET-DNA mediated NFκB activation, we found that VEGF-A was regulated by NFκB activation (Fig. S6D). We further investigated whether NET-DNA stimulated the ITGAV/NFκB pathway to induce VEGF-A biogenesis and angiogenesis. ELISA assay results showed that addition NFκB inhibitor or the knockdown of ITGAV decreased the expression of VEGF-A induced by NET-DNA. (Fig. 6F). Similarly, conditioned medium from CCA cell lines was utilized to conduct in vitro capillary-like tube formation assay, by quantifying the capillary-like tube length, illustrated that inhibition of the ITGAV/NFκB pathway diminished effects of NET-DNA on angiogenesis (Fig. 6G). Collectively, NET-DNA exerted pro-angiogenic activities mainly through activation of ITGAV/ NFκB pathway.

## Discussion

**Fig. 7:**
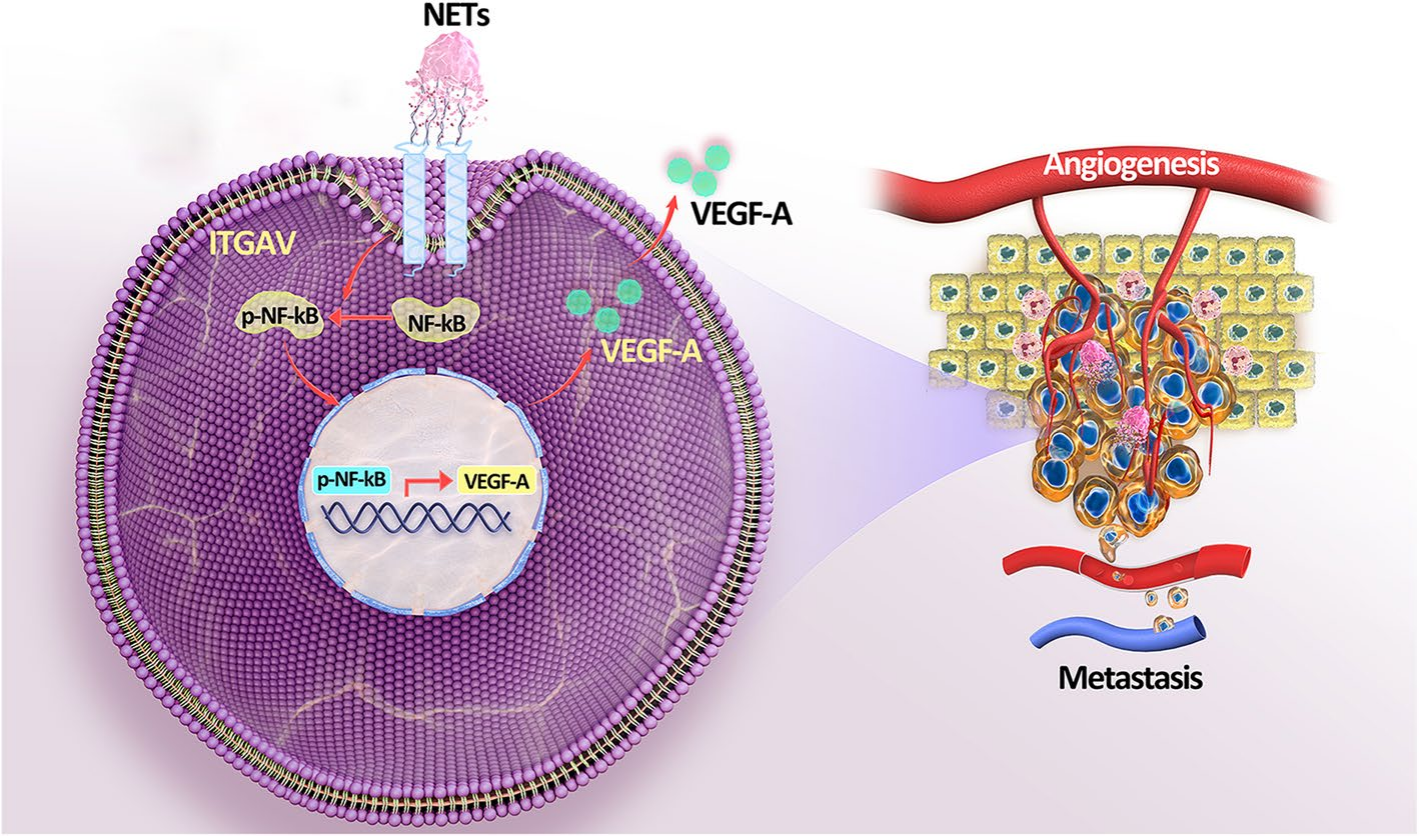
Schematics highlighting the major findings of this study. Increased NETs deposition in tumor microenvironment was responsible for CCA proliferation, metastasis and angiogenesis, which was driven predominantly by interaction with αV integrin and activation of the NFκB pathway

CCA is the second most common primary liver cancer worldwide which originates from the bile duct with an extremely short survival time [18]; hence it is critical to identify the molecular targets and underlying mechanism. In this study, our data indicated that intratumoral increased NETs deposition was responsible for CCA proliferation, metastasis and angiogenesis, which was driven predominantly by interaction with αV integrin and activation of the NFκB pathway (Fig. 7). Activated NFκB directly stimulated VEGF-A transcription via binding to its promoter regulatory region. In our research, via the GEO databases, we found that NETs_score was higher in CCA tissues compared with adjacent non-tumor tissues. Although the number of samples in GEO database of CCA was relatively few, our data showed that a high score of NETs was linked to worse OS and DFS in patients with CCA, which was consistent with a previous study that NETs were associated with poor prognosis in metastatic colorectal cancer, accordingly [19]. Future studies collecting more samples of CCA may further determine the practical value of NETs in predicting prognosis in CCA.

In vitro*,* we verified that NETs stimulation enhanced the proliferation, migration, and invasion abilities of CCA cells. To investigate whether the promoted migration and invasion were linked to cell proliferation, we conducted a CCK8 growth assay, revealing a significant growth difference at least 48 hours after NETs treatment. However, the transwell migration and invasion assays were limited to 18 hours. Furthermore, the CCK8 growth assay indicated that at low concentrations, NETs exhibited pro-inflammatory effects, with the tumor growth rate increasing as NETs concentration rose. Conversely, at high NET concentrations, NETs displayed cytotoxicity, likely inhibiting proliferation. Our data suggested that NETs components exert distinct modes of action. Previous studies have reported the cytotoxicity of histones in vitro, and their release in the circulation causes lethality in mouse models of liver injury [20]. Therefore, we hypothesized that the contribution of NET-DNA in directly facilitating carcinogenesis was limited by the histone cytotoxicity threshold. The cytotoxicity of histones from NETs to CCA cells was planned for investigation in future studies.

On the molecular level, our investigation revealed the role of NET-DNA in regulating EMT via enhancing the expression of the mesenchymal markers vimentin, and decreasing the expression of the epithelial marker E-cadherin during CCA migration and invasion. This involvement of NET-DNA in EMT is dependent on the transcription factor Twist. Besides, NFκB pathways are involved in NET-DNA mediated CCA proliferation and metastasis. A small molecular inhibitor was applied to verify that NFκB pathway activation contributes to the oncogenic effects of NET-DNA in CCA cells. It is also reported that phosphorylation of NFκB could interact with HSP27 resulting in increased the expression of Twist and facilitated radiation-mediated EMT [21]. However, further study is required to elucidate the relationship between p-NFκB and Twist in CCA.

Integrins-dependent mechanisms have been associated with multiple steps of cancer progression and αV integrin is considered a key-molecule in the proliferation, EMT, angiogenesis, and metastasis process in several cancers [22–24]. Notably, a crucial finding of our study was the first evidence that the interaction between NET-DNA and αV integrin was functionally involved in promoting proliferation and metastasis, as well as the activation of NFκB in CCA cells, which shedding light on new mechanisms of CCA development and progression. In addition, we observed a positive correlation between αV integrin and p-NFκB in CCA specimens from our transposon-based model. Therefore, NET-DNA promoted CCA progression, at least in part, by activating the αV integrin/NFκB pathway.

Angiogenesis, the formation of new blood vessels, is a well-established hallmark of cancer and plays a pivotal role in tumor growth and progression. In the case of CCA, inhibition of angiogenesis provides a promising targeted therapeutic option for CCA [25]. The vascular endothelial growth factor (VEGF) is a potent pro-angiogenic factor that holds a crucial role in pathological angiogenesis. Overexpression of VEGF has been identified in CCA and is associated with advanced disease stages and poor prognosis [26]. However, targeting VEGF alone has not improved the prognosis of CCA patients in clinical trials [27]. Here, we found that NET-DNA selectively activated the αV integrin/NFκB pathway to promote CCA cells’ synthesis and release of VEGF-A. We also confirmed that activated NFκB could directly bind to the VEGF-A promoter regulatory region and upregulate VEGF-A expression to stimulate angiogenesis. Moreover, a previous study focusing on the role of integrins in angiogenesis has demonstrated that targeting the αV integrin on HUVEC with Salmonella bacteria delivery vector could inhibit the angiogenesis process in cancer therapy [28]. Thus, we predict that NET-DNA can not only communicate with CCA cells, but also interact with vascular endothelial cells to facilitate angiogenesis and this issue needs further investigation. Our investigation into the role of NETs in promoting angiogenesis in CCA aims to provide a theoretical basis for targeted therapy involving NETs in combination with anti-angiogenesis for treating CCA.

## Conclusions

We found that CCA tissues induced the formation of NETs, which favor tumor growth and progression. More importantly, the precise component of the NETs structure that interacts with tumor cells and the nature of the downstream pathway were elucidated, thereby NET-DNA targeted therapies may thus be a promising therapeutic avenue that can enhance the efficacy of immunotherapy. Studies have demonstrated that the degradation of NETs by DNase-I resulted in the destruction of their web-like structure and loss of capacity to promote metastasis. In brief, the direct effect of NET-DNA on tumor cells implies that it can be targeted in novel therapies to combat CCA metastasis and recurrence.

### Novelty and impact

The study unequivocally establishes NETs as facilitators of CCA progression, orchestrating proliferation, metastasis, and angiogenesis through ITGAV/NFκB pathway activation. This novel insight positions NETs as prospective therapeutic targets for managing CCA patients.

### Supplementary Information

**Additional file 1:** **Supplementary Figure 1.** NETs were induced in vitro. (A) HE staining of neutrophils from health volunteers. Scale bar=60μm. (B) Representative images of MPO and CitH3 staining in the NETs induced by PMA or normal neutrophils. Scale bar=10μm.

**Additional file 2:** **Supplementary Figure 2.** The DNA of NETs promoted CCA cells proliferation, migration, and invasion. (A) Proliferation rate of CCA cells cocultured with indicated NETs components. (B) Transwell migration and invasion assays of CCA cells cocultured with indicated NETs components. (C) Migration assays for RBE cells stimulated with NET-DNA at increasing concentrations (0-7.5μg/ml) or pretreated with DNase-I (0.25mg/ml). (D) Representative immunofluorescence images of E-cadherin and vimentin expression in indicated CCA cell lines treated with or without NET-DNA, (5ng/μl, 24h) Scale bar=15μm. (E) qPCR analyze the expression of Slug and Snail in RBE and KMBC cells treated with NET-DNA or vehicle (5ng/μl, 24h). ***P<0.001. Data are means ± SD of three independent experiments.

**Additional file 3:** **Supplementary Figure 3.** NETs formation was inhibited in PAD4 KO transposon-based model mice. (A) Agarose gel electrophoresis was applied to confirm the genotype of the mice. (B) Immunofluorescence detection of MPO and CitH3 in the WT mice and PAD4 KO mice, Scale bar=30μm.

**Additional file 4:** **Supplementary Figure 4.** NET-DNA promoted the progression of CCA via NKκB signaling pathway. (A) Gene set enrichment analysis showed that NFκB signaling pathway was enriched in NETs treatment group. (B) Correlation of expression of p-NFκB and tumor size in WT (*n*=45, *p*<0.001, Spearman’s coefficient r=0.61) and PAD4KO (*n*=45, *p*=0.04, Spearman’s coefficient *r*=0.31) CCA mice. (C) Transwell migration and invasion assays for indicated cell lines. ***P<0.001. Data are means ± SD of three independent experiments.

**Additional file 5:** **Supplementary Figure 5.** ITGAV was recognize as the potential DNA receptor. (A) Mass spectrometry analysis the protein interacted with NET-DNA for three times. (B) Sequence alignment of the ITGAV with DNA-binding domains of a classical DNA sensor HMGB1. (C) Relative ITGAV expression levels in 36 CCA and 9 normal samples from The Cancer Genome Atlas database. (D) qPCR results indicated that shITGAV-3 exhibited the greatest silencing effect. (E) Immunofluorescence indicated that shRNA inhibited ITGAV expression. (F) IF staining of ITGAV transfected RBE treated with NET-DNA showing downregulated expression of p-NFκB. Dapi (blue), p-NFκB (red), tubulin (green), Scale bar=50μm. *P<0.05, ***P<0.001. Data are means ± SD of three independent experiments.

**Additional file 6:** **Supplementary Figure 6.** NET-DNA promoted CCA cell lines synthesized VEGF-a via ITGAV/ NFκB axis and induced angiogenesis. (A) Heat meap of angiogenesis-related genes in DEGs. (B) Gene set enrichment analysis showed that VEGF signatures were enriched in NETs treatment group. (C) Correlation of protein expression of CD34 and tumor size (*n*=45, *p*=0.2, Spearman’s coefficient r=0.18). (D) qPCR was applied to verified that VEGF-A biogenesis in CCA cells was regulated by p-NFκB. ***P<0.001. Data are means ± SD of three independent experiments.

**Additional file 7.**

**Additional file 8.**

## Data Availability

The datasets used and/or analyzed during the current study are available from the corresponding author upon reasonable request.

## Abbreviations

CCA: cholangiocarcinoma
NETs: neutrophil extracellular traps
ITGAV: integrin αV
NFκB: nuclear factor kappa-B
HC: immunohistochemical
IF: immunofluorescence
ChIP: Chromatin immunoprecipitation
MPO: myeloperoxidase
TGFβ: transforming growth factor betta
DMSO: dimethyl sulfoxide
DAPI: 4′,6-diamidino-2-phenylindole;
