# Supplementary figures and images for: The scaffold of neutrophil extracellular traps promotes CCA progression and modulates angiogenesis via ITGAV/NFκB
Source: Cell Commun Signal. 2024 Feb 8;22:103. doi: 10.1186/s12964-024-01500-5 (PMC10851487; doi:10.1186/s12964-024-01500-5)

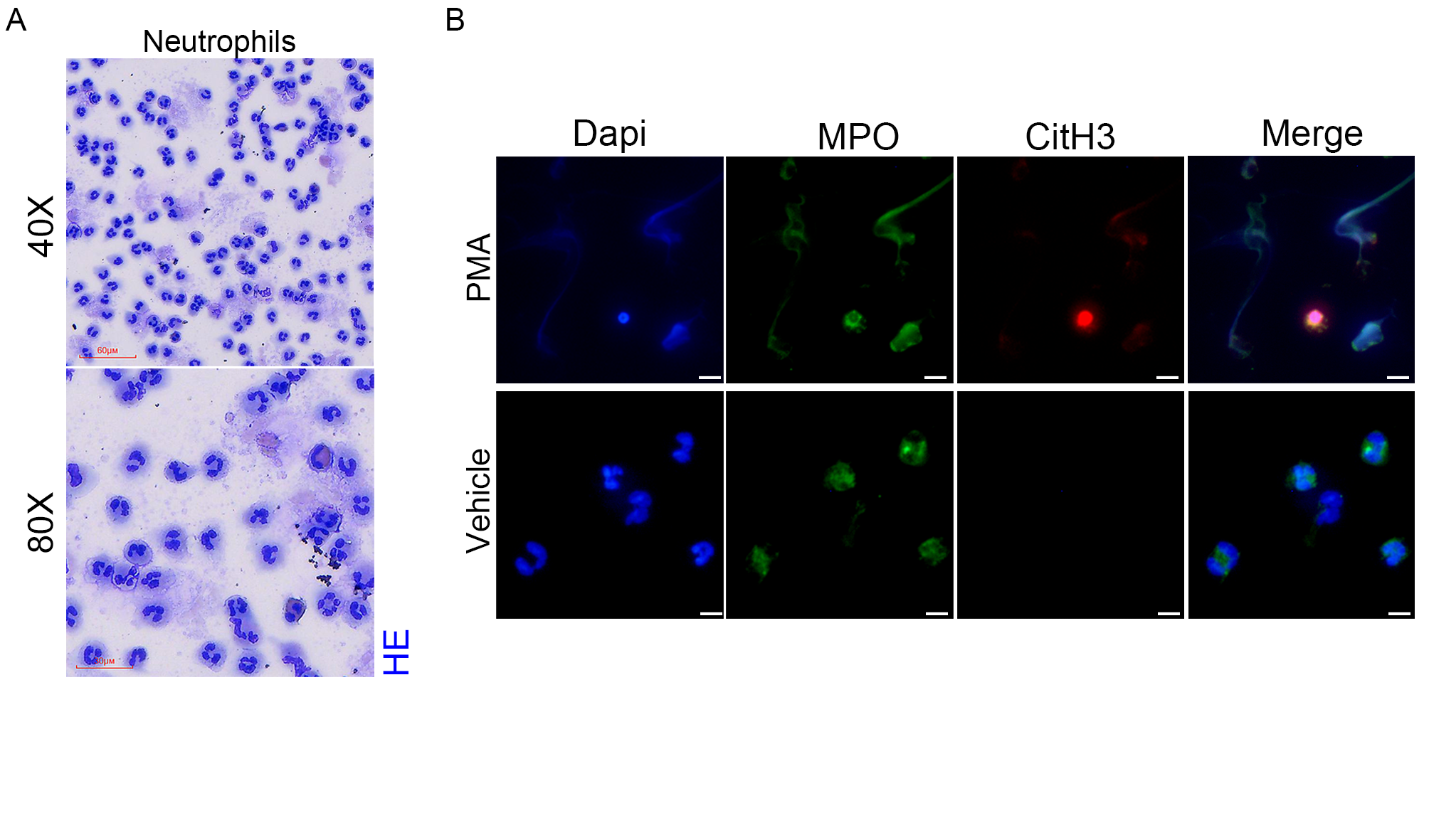

Supplement: Supplementary file 1 — Additional file 1: Supplementary Figure 1. NETs were induced in vitro. (A) HE staining of neutrophils from health volunteers. Scale bar=60μm. (B) Representative images of MPO and CitH3 staining in the NETs induced by PMA or normal neutrophils. Scale bar=10μm. [file 12964_2024_1500_MOESM1_ESM.tif]

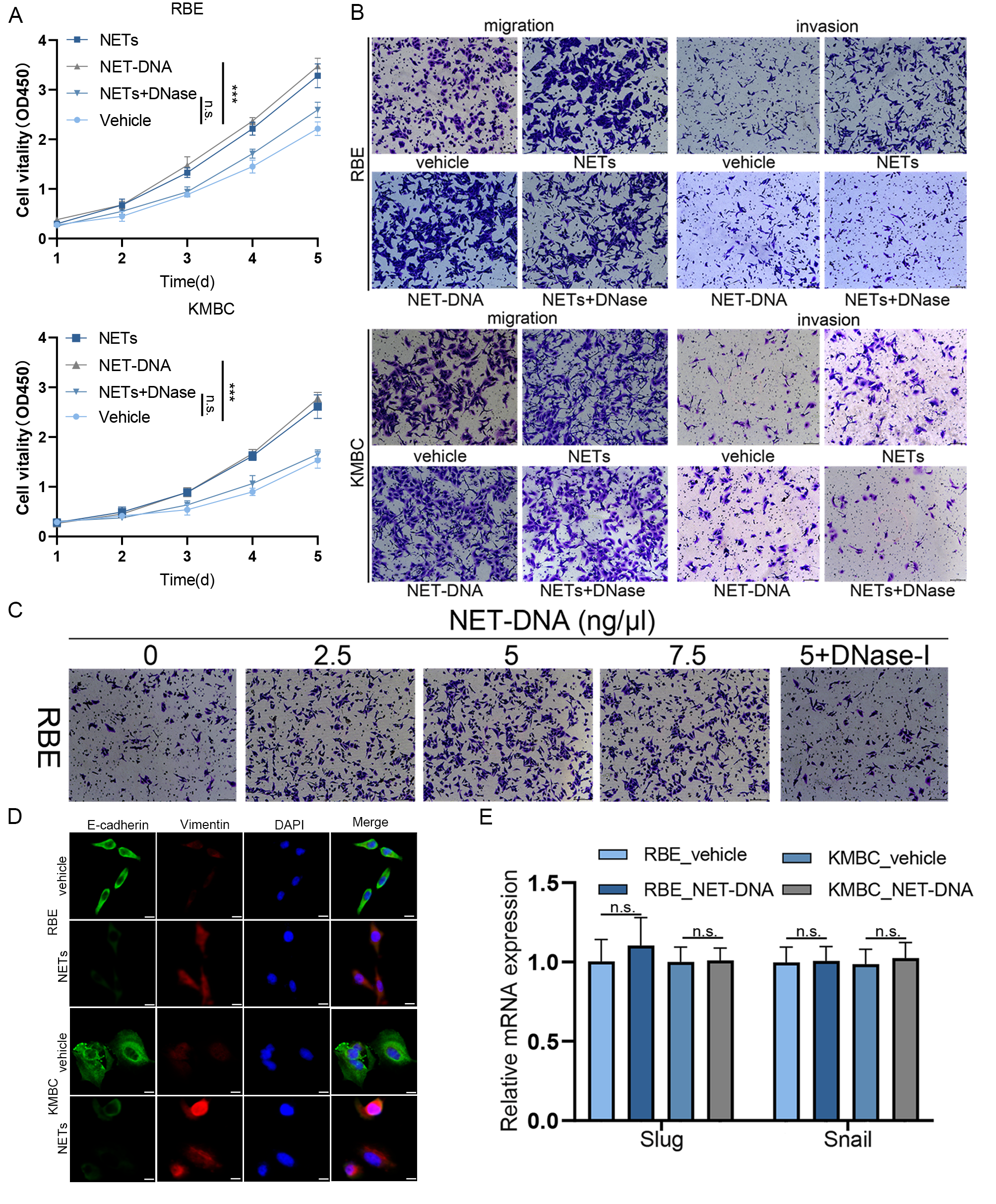

Supplement: Supplementary file 2 — Additional file 2: Supplementary Figure 2. The DNA of NETs promoted CCA cells proliferation, migration, and invasion. (A) Proliferation rate of CCA cells cocultured with indicated NETs components. (B) Transwell migration and invasion assays of CCA cells cocultured with indicated NETs components. (C) Migration assays for RBE cells stimulated with NET-DNA at increasing concentrations (0-7.5μg/ml) or pretreated with DNase-I (0.25mg/ml). (D) Representative immunofluorescence images of E-cadherin and vimentin expression in indicated CCA cell lines treated with or without NET-DNA, (5ng/μl, 24h) Scale bar=15μm. (E) qPCR analyze the expression of Slug and Snail in RBE and KMBC cells treated with NET-DNA or vehicle (5ng/μl, 24h). ***P<0.001. Data are means ± SD of three independent experiments. [file 12964_2024_1500_MOESM2_ESM.tif]

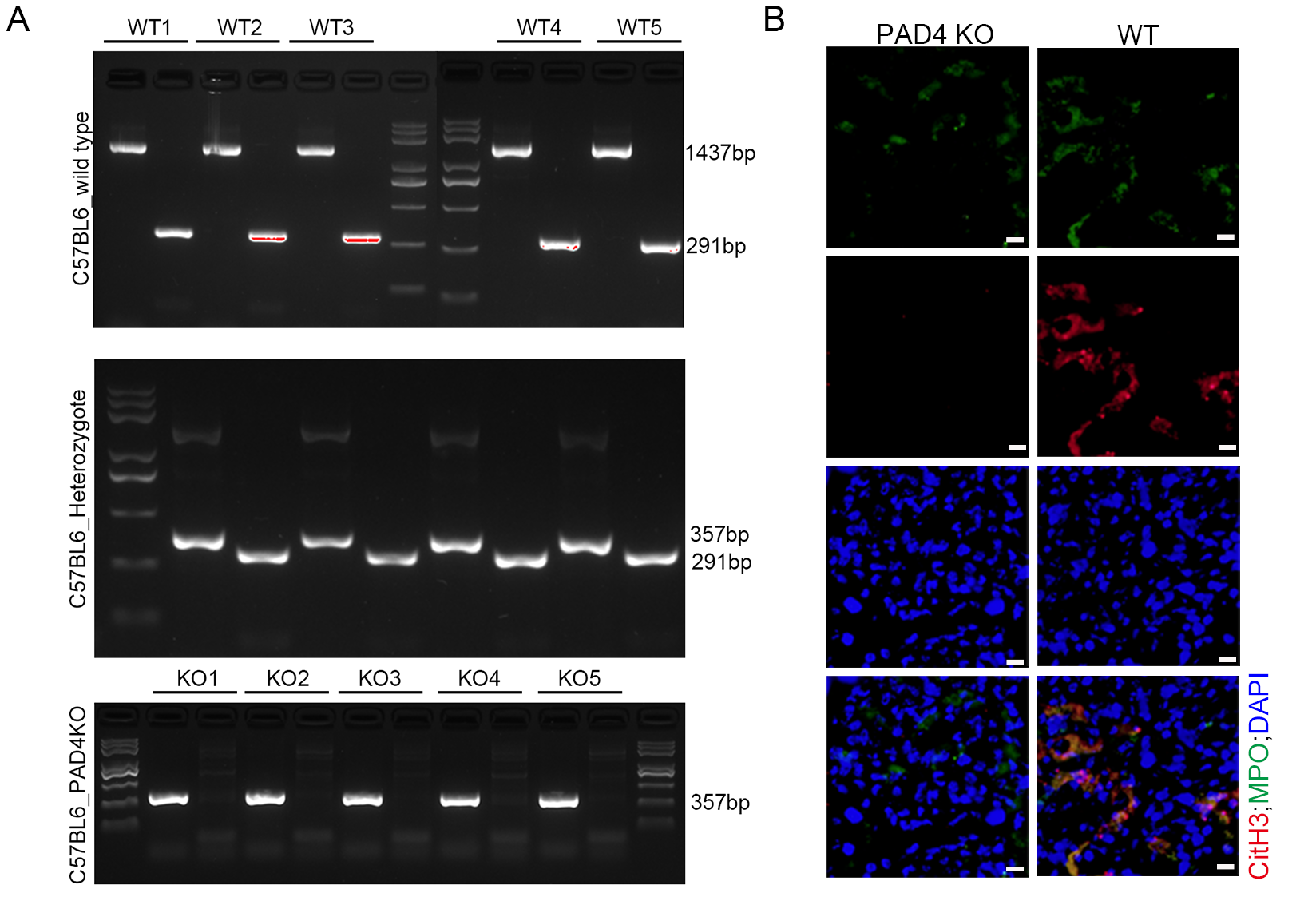

Supplement: Supplementary file 3 — Additional file 3: Supplementary Figure 3. NETs formation was inhibited in PAD4 KO transposon-based model mice. (A) Agarose gel electrophoresis was applied to confirm the genotype of the mice. (B) Immunofluorescence detection of MPO and CitH3 in the WT mice and PAD4 KO mice, Scale bar=30μm. [file 12964_2024_1500_MOESM3_ESM.tif]

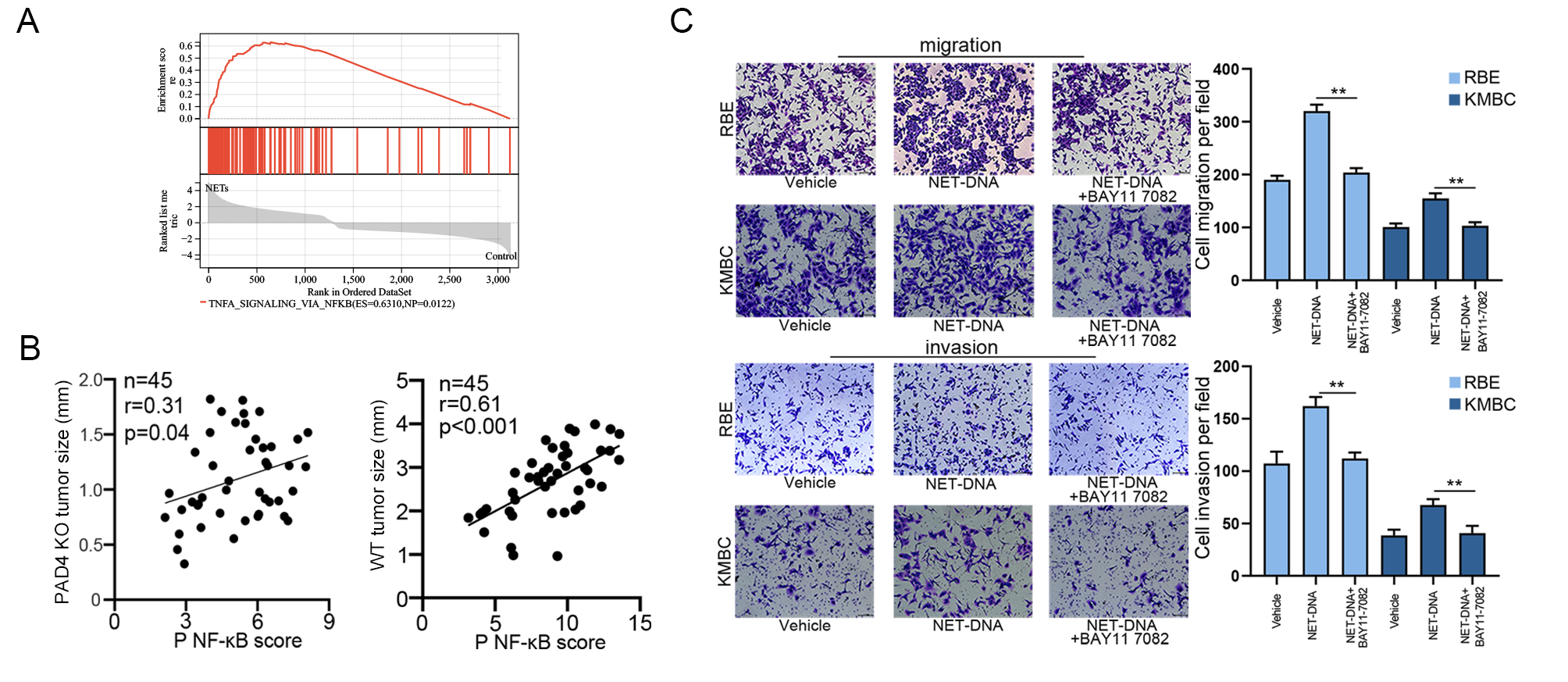

Supplement: Supplementary file 4 — Additional file 4: Supplementary Figure 4. NET-DNA promoted the progression of CCA via NKκB signaling pathway. (A) Gene set enrichment analysis showed that NFκB signaling pathway was enriched in NETs treatment group. (B) Correlation of expression of p-NFκB and tumor size in WT (n=45, p<0.001, Spearman’s coefficient r=0.61) and PAD4KO (n=45, p=0.04, Spearman’s coefficient r=0.31) CCA mice. (C) Transwell migration and invasion assays for indicated cell lines. ***P<0.001. Data are means ± SD of three independent experiments. [file 12964_2024_1500_MOESM4_ESM.tif]

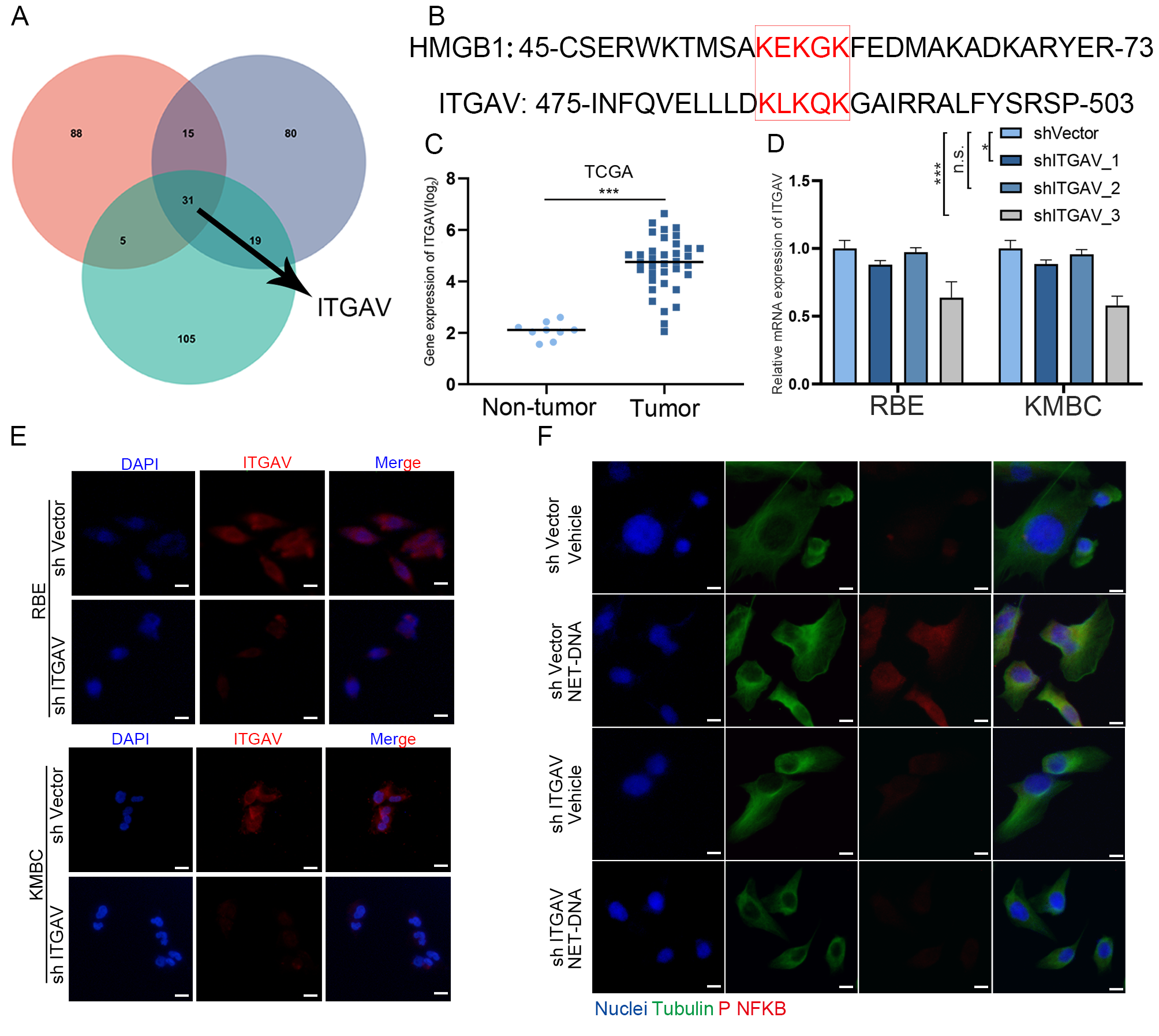

Supplement: Supplementary file 5 — Additional file 5: Supplementary Figure 5. ITGAV was recognize as the potential DNA receptor. (A) Mass spectrometry analysis the protein interacted with NET-DNA for three times. (B) Sequence alignment of the ITGAV with DNA-binding domains of a classical DNA sensor HMGB1. (C) Relative ITGAV expression levels in 36 CCA and 9 normal samples from The Cancer Genome Atlas database. (D) qPCR results indicated that shITGAV-3 exhibited the greatest silencing effect. (E) Immunofluorescence indicated that shRNA inhibited ITGAV expression. (F) IF staining of ITGAV transfected RBE treated with NET-DNA showing downregulated expression of p-NFκB. Dapi (blue), p-NFκB (red), tubulin (green), Scale bar=50μm. *P<0.05, ***P<0.001. Data are means ± SD of three independent experiments. [file 12964_2024_1500_MOESM5_ESM.tif]

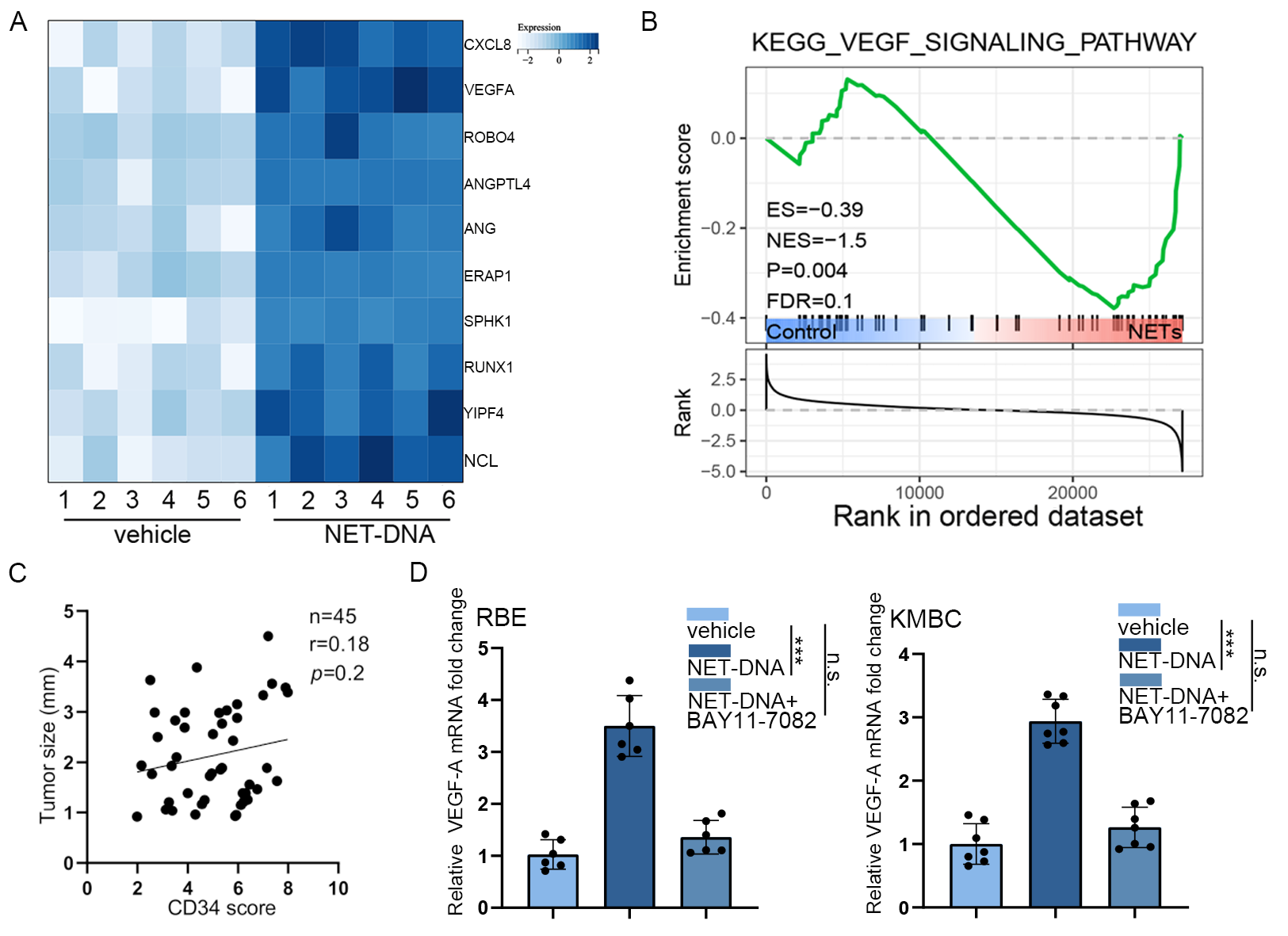

Supplement: Supplementary file 6 — Additional file 6: Supplementary Figure 6. NET-DNA promoted CCA cell lines synthesized VEGF-a via ITGAV/ NFκB axis and induced angiogenesis. (A) Heat meap of angiogenesis-related genes in DEGs. (B) Gene set enrichment analysis showed that VEGF signatures were enriched in NETs treatment group. (C) Correlation of protein expression of CD34 and tumor size (n=45, p=0.2, Spearman’s coefficient r=0.18). (D) qPCR was applied to verified that VEGF-A biogenesis in CCA cells was regulated by p-NFκB. ***P<0.001. Data are means ± SD of three independent experiments. [file 12964_2024_1500_MOESM6_ESM.tif]

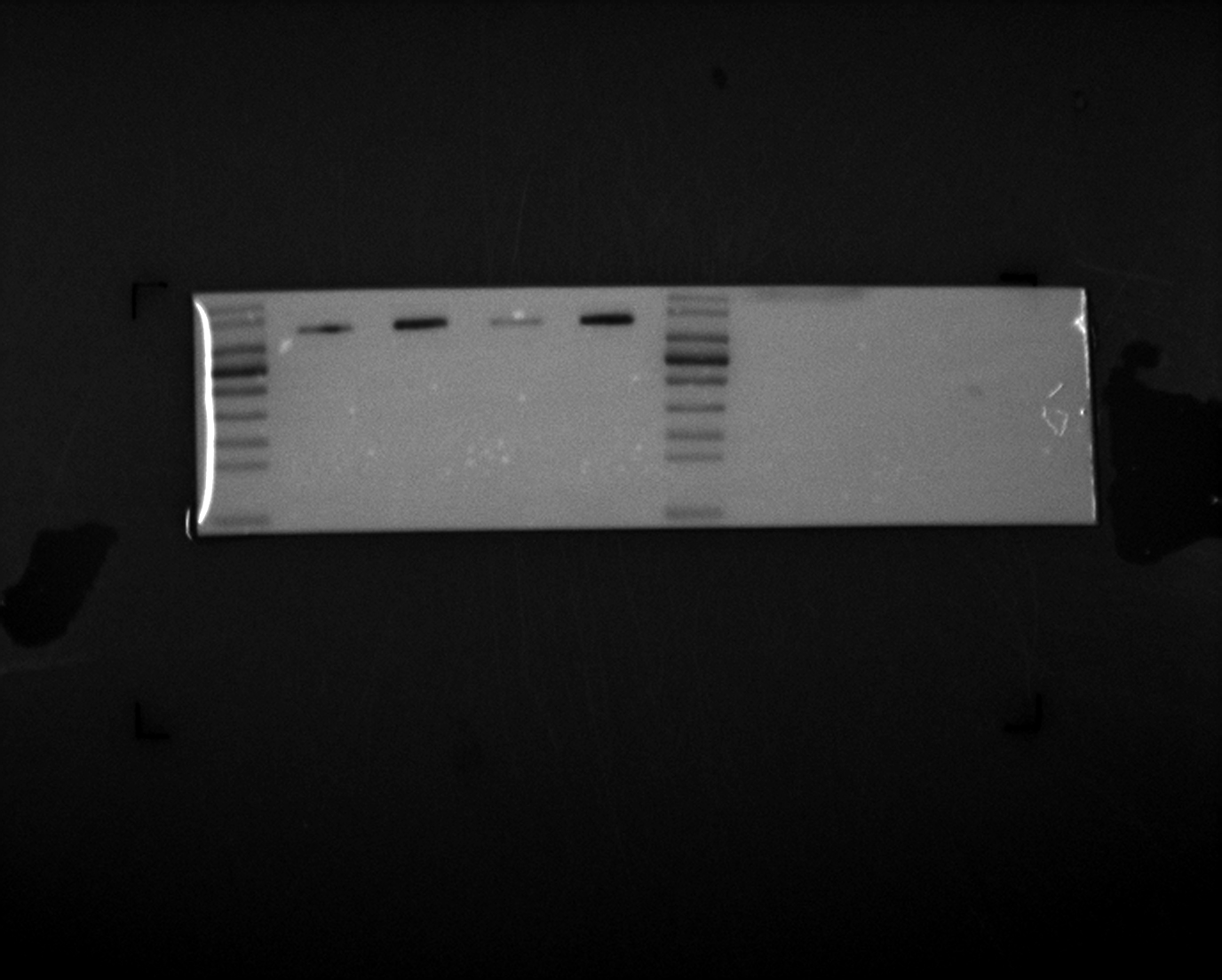

Supplement: Supplementary file 7 — Additional file 7. [file 12964_2024_1500_MOESM7_ESM.tif]
