# Supplementary material for: The scaffold of neutrophil extracellular traps promotes CCA progression and modulates angiogenesis via ITGAV/NFκB
Source: Cell Commun Signal. 2024 Feb 8;22:103. doi: 10.1186/s12964-024-01500-5 (PMC10851487; doi:10.1186/s12964-024-01500-5)
Supplement: Supplementary file 8 — Additional file 8. [file 12964_2024_1500_MOESM8_ESM.docx]

Table1. CCA patients clinical characteristics

| Patients_ID | Patients_Operation Time | Patients_Age | Patients_Gender | Patients_TNM Stage | Patients_Pathological Diagnosis |
| --- | --- | --- | --- | --- | --- |
| 191903CH | 2019-7-29 | 48 | Female | stage: II | Tumor (Intrahepatic cholangiocarcinoma) |
| 191930CH | 2019-7-29 | 63 | Female | stage: III | Tumor (Intrahepatic cholangiocarcinoma) |
| 192061CH | 2019-8-14 | 65 | Male | stage: I | Tumor (Intrahepatic cholangiocarcinoma) |
| 192090CH | 2019-8-23 | 61 | Female | stage: II | Tumor (Intrahepatic cholangiocarcinoma) |
| 192092CH | 2019-8-26 | 66 | Female | stage: I | Tumor (Intrahepatic cholangiocarcinoma) |
| 192276CH | 2019-9-10 | 53 | Female | stage: I | Tumor (Intrahepatic cholangiocarcinoma) |
| 192572CH | 2019-10-30 | 72 | Male | stage: III | Tumor (Intrahepatic cholangiocarcinoma) |

Table2. Primary antibodies for WB, IHC and IF

| Antibody | Concentration  for WB | Concentration  for IHC | Concentration  for IF | Specificity | Catalog |
| --- | --- | --- | --- | --- | --- |
| CitH3 | 1:1000 | 1:100 | 1:100 | Rabbit polyclonal | Ab5103 |
| MPO |  |  | 1:100 | Mouse monoclonal | 66177-1-Ig |
| E-cadherin | 1:1000 |  | 1:100 | Mouse monoclonal | Ab231303 |
| Vimentin | 1:2000 |  | 1:200 | Rabbit polyclonal | Ab92547 |
| Snail | 1:1000 |  |  | Rabbit monoclonal | Ab216347 |
| Slug | 1:1000 |  |  | Rabbit polyclonal | CST 9585S |
| Twist | 1:1000 |  |  | Mouse monoclonal | Ab50887 |
| NFκB | 1:1000 |  |  | Rabbit monoclonal | CST 8242S |
| p-NFκB | 1:1000 | 1:100 | 1:100 | Rabbit monoclonal | CST 3033S |
| ITGAV | 1:1000 | 1:100 | 1:100 | Mouse monoclonal | Ab179475 |
| CD34 |  | 1:100 |  | Rabbit polyclonal | Ab81289 |
| Tubulin |  |  | 1:100 | Mouse monoclonal | Ab7291 |
| Ki-67 |  | 1:200 |  | Rabbit polyclonal | Ab16667 |
| VEGF-A | 1:1000 |  |  | Rabbit polyclonal | Ab46154 |

Table3. Sequence of Primers for PCR

| Primers |  | Sequences (5’------3’) |
| --- | --- | --- |
| E-cadherin | Forward： | 5’-TTGCTACTGGAACAGGGACAC-3’ |
|  | Reverse： | 5’-GATGTATTGGGAGGAAGGTCTG-3’ |
| Vimentin | Forward： | 5’-TTGAACGCAAAGTGGAATC-3’ |
|  | Reverse： | 5’-AGGTCAGGCTTGGAAACA-3’ |
| Twist | Forward： | 5’-CGACGACAGCCTGAGCAACA-3’ |
|  | Reverse： | 5’-CCACAGCCCGCAGACTTCTT-3’ |
| Snail | Forward： | 5’-TTTCTGGTTCTGTGTCCTCTG-3’ |
|  | Reverse： | 5’-TGTCAGCCTTTGTCCTGTAGC-3’ |
| Slug | Forward： | 5’-CCTCCATCTGACACCTCC-3’ |
|  | Reverse： | 5’-TGCAACGCGAGTCTGTGTTT-3’ |
| ITGAV | Forward： | 5’-GTTTCAGTGTGCACCAGCAG-3’ |
|  | Reverse： | 5’-AAGGCCACTGAAGATGGAGC-3’ |
| GAPDH | Forward： | 5’-GCACCGTCAAGGCTGAGAAC-3’ |
|  | Reverse： | 5’-TGGTGAAGACGCCAGTGGA-3’ |
| VEGF-A | Forward： | 5’-CCTCCATCTGACACCTCC-3’ |
|  | Reverse： | 5’-TGCAACGCGAGTCTGTGTTT-3’ |

Table4. Related gene sets calculated for Z-score

| NETs NETs related gene sets |
| --- |
| NETs_score CYP4F3, SLC22A4, IL17A, MPO, F3, TNFRSF10C, CREB5, SELP, VNN3, MME, ENTPD4, G0S2, S100A12, BST1, CLEC6A, HPSE, FPR2, CEACAM3, IL8 |

| Angiogenesis Angiogenesis related gene sets |
| --- |
| Angiogenesis_score EMCN, HTATIP2, ACVRL1, IL18, RNH1, PML, PF4, TNFSF12, FOXO4, CANX, SPINK5, SHH, TGFB2, AGGF1, ANG, L17F, ROBO4, RHOB, ERAP1, CHRNA7, RUNX1, EGF, ANGPTL3, C1GALT1, ANGPTL4, SCG2, COL4A3, COL4A2, CXCL8, SPHK1, NF1, NPR1, MYH9, TNNI3, NCL, PLG, THY1, PROK2, CDH13, SERPINF1, EPGN, STAB1, BTG1, NOTCH4, VEGFA, AMOT, NPPB, ATP5IF1 |
